# Transcriptome analyses of the human retina identify unprecedented transcript diversity and 3.5 Mb of novel transcribed sequence via significant alternative splicing and novel genes

**DOI:** 10.1186/1471-2164-14-486

**Published:** 2013-07-18

**Authors:** Michael H Farkas, Gregory R Grant, Joseph A White, Maria E Sousa, Mark B Consugar, Eric A Pierce

**Affiliations:** 1 Ocular Genomics Institute, Department of Ophthalmology, Massachusetts Eye and Ear Infirmary, Harvard Medical School, Boston, MA, USA; 2 Berman-Gund Laboratory for the Study of Retinal Degenerations, Department of Ophthalmology, Massachusetts Eye and Ear Infirmary, Harvard Medical School, Boston, MA, USA; 3 Penn Center for Bioinformatics, University of Pennsylvania, Philadelphia, PA, USA

**Keywords:** RNA-Seq, Transcriptome, Inherited retinal degeneration, Retina, Novel genes, Alternative splicing

## Abstract

**Background:**

The retina is a complex tissue comprised of multiple cell types that is affected by a diverse set of diseases that are important causes of vision loss. Characterizing the transcripts, both annotated and novel, that are expressed in a given tissue has become vital for understanding the mechanisms underlying the pathology of disease.

**Results:**

We sequenced RNA prepared from three normal human retinas and characterized the retinal transcriptome at an unprecedented level due to the increased depth of sampling provided by the RNA-seq approach. We used a non-redundant reference transcriptome from all of the empirically-determined human reference tracks to identify annotated and novel sequences expressed in the retina. We detected 79,915 novel alternative splicing events, including 29,887 novel exons, 21,757 3′ and 5′ alternate splice sites, and 28,271 exon skipping events. We also identified 116 potential novel genes. These data represent a significant addition to the annotated human transcriptome. For example, the novel exons detected increase the number of identified exons by 3%. Using a high-throughput RNA capture approach to validate 14,696 of these novel transcriptome features we found that 99% of the putative novel events can be reproducibly detected. Further, 15-36% of the novel splicing events maintain an open reading frame, suggesting they produce novel protein products.

**Conclusions:**

To our knowledge, this is the first application of RNA capture to perform large-scale validation of novel transcriptome features. In total, these analyses provide extensive detail about a previously uncharacterized level of transcript diversity in the human retina.

## Background

The human retina is composed of a complex set of cell types. It is estimated that this includes at least 60 types of cells spread across 6 cell classes including: photoreceptor, horizontal, bipolar, amacrine, glial and ganglion cells [1,2]. Its normal function is dependent upon each cell type working properly in a coordinated fashion. Multiple disorders affect the retina and cause vision loss, including diabetes, age-related macular degeneration, inherited retinal degenerations (IRDs) and cancer, which are common causes of vision loss in patients of all ages [3-6]. While research has lead to the development of effective therapies for some of these disorders, such as the successful application of gene augmentation therapy to treat the severe, early onset form of inherited retinal degeneration (IRD) Leber congenital amaurosis (LCA) caused by mutations in the *RPE65* (ENSG00000116745) gene [7-9], therapies for many types of retinal disease remain to be developed.

Sequencing of the 191 known IRD disease genes in patients with recessive IRDs can result in the identification of a single mutant allele, but fail to identify a second mutation [10,11]. Since these re-sequencing efforts are focused on annotated exons, it is possible that unidentified transcribed sequences harbor some of the missing mutations [12-14]. For example, identification of novel exons in the *BBS8* (ENSG00000165533) and *RPGR* (ENSG00000156313) genes lead to the detection of additional disease causing mutations [12,13]. Specifically, the inclusion of exon 2a in *BBS8* is a retina-specific alternative splicing event [12]. Mutations in *BBS8* cause Bardet-Biedl syndrome, a syndromic form of IRD, characterized by rod-cone dystrophy, obesity, and polydactyly, among other phenotypic disorders [15,16]. An in-frame mutation in the splice acceptor site results in skipping of exon 2A and leads to the non-syndromic form of retinitis pigmentosa (RP) [12]. A retina-specific novel exon 15a in *RPGR* contains a stop codon and leads to a protein that is 55–169 amino acids shorter than in other tissues, which was found to be required for normal retinal function [13]. Mutations in this ORF15 are a common cause of X-linked RP [17].

RNA-seq is a powerful method for studying the transcriptional landscape of a given cell or tissue. Unlike microarrays, RNA-Seq is not limited to current annotations of the transcriptome, allowing for the detection of novel splicing events, including novel genes [18-21]. To date, tens of thousands of novel alternative splicing events and hundreds of novel genes have been identified in a variety of cell and tissue types by RNA-Seq analyses [18,22,23]. A unique feature of RNA-Seq libraries generated from poly-A RNA is the ability to detect certain types of non-coding RNAs (ncRNAs), particularly long intergenic non-coding RNAs (lincRNAs) [24]. lincRNAs resemble protein-coding transcripts in that they are polyadenylated, typically contain multiple exons, and are alternatively spliced, containing 2.3 isoforms, on average. Functionally they are not well characterized, but lincRNAs are known to have important roles in X chromosome inactivation, imprinting, maintaining pluripotency, and regulation of transcription [25]. While not fully studied, over 9,000 lncRNAs (of which lincRNAs are a subgroup) have been identified, and this number is expected to increase substantially, given their high degree of tissue-specificity [24,26].

An analysis of the transcriptome of the human retina using EST data was first reported in 2000 [27]. This work was followed by studies using additional techniques aimed at identifying the genes that were specific to the retina [28-33]. These initial studies increased our understanding of the normal function of the retina, and identified genes involved in the pathogenesis of disease. We have used RNA-Seq to more comprehensively interrogate the human retinal transcriptome. The increased depth of sampling provided by the RNA-Seq approach lead to the identification of 79,915 novel alternative splicing events and over a hundred potential novel genes. Using a targeted enrichment RNA capture approach, we performed a technical validation of 14,696 (18%) of the novel splicing events found in the human retinal transcriptome data. To our knowledge, this is the first application of targeted RNA capture to perform large-scale validation of novel transcriptome features. This method showed that 99% of the putative novel events are real. Bioinformatic analyses indicate that between 15-36% of novel splicing events maintain an open reading frame, and likely result in novel protein-coding transcript isoforms. These analyses also identified 116 putative novel genes. We validated the expression of the full-length gene for 10 of these using independent RT-PCR analyses. These data provide an unprecedented level of information regarding the human retinal transcriptome.

## Results

### Characterization of the annotated retinal transcriptome

We generated RNA-Seq libraries from 3 normal adult human retinal total RNA samples using an adaptation of a standard mRNA-Seq library preparation protocol [34]. The resulting libraries were sequenced using an Illumina HiSeq 2000 instrument. In total, we generated 314 million paired-end sequence reads that were 101 bp in length. We aligned the reads to the human genome (hg19) using the RNA-Seq Unified Mapper (RUM) [35]. RUM aligns RNA-Seq reads in a two-step manner. The first step aligns reads using Bowtie against a reference genome and transcriptome [36]. Reads that do not align in this first step are then aligned to the reference genome using BLAT [37]. Due to the nature of our experiment, aiming to catalog both the annotated human retinal transcriptome as well as novel features, we chose to align our data without preference to a given set of transcriptome annotation tracks. Using this approach, we were able to align nearly 292 million reads (93%), of which 280 million aligned uniquely (89%) (Table 1) (GEO accession - GSE40524).

**Table 1:** Alignment statistics from RUM analysis with no reference transcriptome

	**# of reads**	**% of total reads**
**Total reads**	314,649,719	n.a.
**Uniquely aligned**	280,138,816	89
**Non**-**uniquely aligned**	11,695,005	4
**Total aligned**	291,833,821	93

Currently, 12 annotation reference tracks exist for the human transcriptome in the UCSC genome browser [38]. Eight of these (UCSC, Refseq, CCDS, Vega, Ensembl, Aceview, Gencode, and LincRNAs) are based on empirically-observed EST or RNA-Seq data [24,39-45]. The remaining four annotation tracks (N-Scan, Genscan, SGP, and GeneID) are algorithm-based, generated by scanning the genome for transcription start/stop sites, splice junction signals, etc. [46-50]. Many studies that aim to characterize novel features often do so with only a subset of these annotation tracks [19,20,23,51-53]. For our study, we chose an inclusive approach and built a non-redundant reference transcriptome from the 8 empirically-determined annotation tracks. This reference transcriptome consists of 412,785 unique transcripts (unique CDS start and stop) and over 1 million exons (Additional file 1). We chose not to include the algorithm-based annotation tracks because of their unknown biological relevance.

Using RPKM values, the distribution of transcript abundance was determined. Approximately, 97% of the transcripts had an RPKM value less than 100, with the most highly expressed transcripts having an RPKM value greater than 10,000 (Additional file 2). Using the standard of 1–4 RPKM being equal to one transcript/cell, this suggests that we have detected between 1 to 2500 transcripts, at a minimum per cell [54]. Approximately 50% of all expressed transcripts fall within the 5–25 RPKM (5–25 transcripts/cell) range. As shown in Additional file 2, the distribution of expression levels is very similar between the three retinal RNA samples, with an overall concordance of 91%. The most highly expressed transcripts correspond to proteins involved in mitochondrial respiration with at least 2500 transcripts/cell, which is not surprising given the high metabolic demand of the retina.

**Figure 1:**
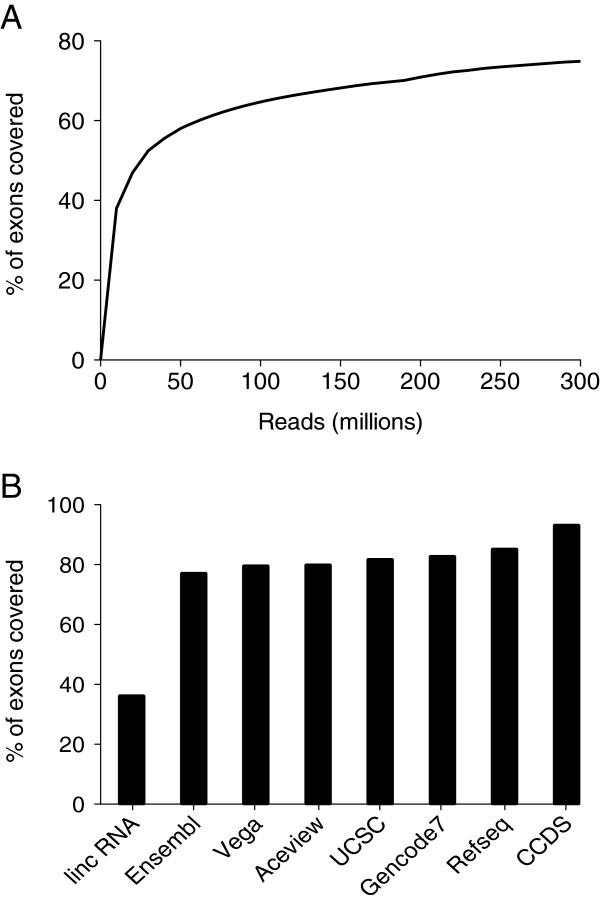
**Depth of coverage plots for detected exons found in a non**-**redundant database created from the eight empirically determined human annotation tracks.** This database included a total of 1,016,490 unique annotated exons. **A)** Depth of coverage determined from the combined set of annotation tracks by detecting the number of annotated exons with an average of 1 and 5 read coverage at every 10 million reads. As shown, the plots approach plateaus at 300 million reads, suggesting the transcriptome is nearly fully covered at this level of sequencing. **B)** Depth of coverage for the individual annotation tracks at 300 million reads. The lincRNA and CCDS annotation tracks have the lowest (35%) and highest (94%), respectively. On average, the other 6 annotation tracks are covered near 80%.

At 314 million reads, we were able to detect 75% of all exons annotated in the reference transcriptome at an average read depth of 5 or greater (Figure 1A). Some additional exons can be observed at average read depth of 1 or greater, with 83% of all exons detected, corresponding to approximately 160,000 unique transcripts. Detection of annotated exons from the standpoint of the individual databases was relatively consistent, aside from the lincRNA and CCDS databases (Figure 1B). We detected only 35% of the annotated lincRNA exons, but 90% of the annotated CCDS exons, at an average read depth of 5. We detected between 77-82% of the annotated exons in the remaining 6 databases.

### Visualization of the retinal transcriptome

The coverage and splice junction data for the human retinal transcriptome can be viewed from our website at http://oculargenomics.meei.harvard.edu/index.php/ret-trans/110-human-retinal-transcriptome. Additional file 3 is a representative image of our data with a detailed description of each feature. These data provide the ability to interrogate genes of interest for expression of individual isoforms, novel features, and the relative abundance for both the annotated and novel features.

### A significant fraction of detected splice junctions are novel

We compared our aligned data to the eight empirically-determined annotation tracks as previously mentioned. We found that a total of 825,781 reads crossed 117,030 novel splice junctions, while a total of 105,911,729 million reads crossed 229,906 annotated splice junctions (Table 2). While this number of junctions is nearly identical to that detected other transcriptome studies, it is fewer than the 438,000 junctions predicted to correspond to the transcripts detected [54]. This is not surprising given that nearly 50% of the detected transcripts have an RPKM level of less than 5, and only one out of every three reads will cross a splice junction. We have previously calculated that the false positive/false negative rates for detection of splice junctions using the RUM analysis algorithm are 1.41% and 2.48%, respectively suggesting that the majority of the novel junctions detected are real [35].

**Table 2:** Statistics for reads crossing splice junctions

**Junction type**	**# of Reads**	**# of Junctions**
**Annotated**	105,911,729	229,906
**Novel**	825,781	117,030

**Figure 2:**
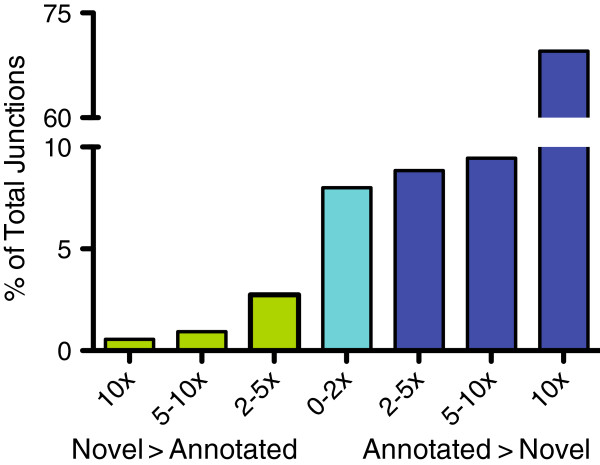
**The ratio of novel to annotated isoform abundance as determined by reads crossing splice junctions.** The number of reads crossing a novel splice junction relative to the corresponding annotated splice junction were used to calculate the ratio. This data demonstrates that a relatively significant portion (~15%) of novel isoforms in the human retinal transcriptome are at least as abundant or more abundant than their annotated counterparts. The green bars represent the percentage of total junctions in which the novel isoform was more abundant than the annotated. The blue-green bar represents the percentage of total junctions where the novel and annotated isoforms are equal (< 2-fold change). The blue bars represent the percentage of total junctions in which the annotated isoforms are more abundant than the novel.

**Figure 3:**
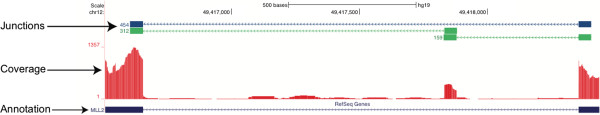
***MLL2 *is a disease-relevant gene in which a novel exon that constitutes a major transcript isoform was detected.** The scale is provided by the bar at the top of the figure. The numbers shown indicate position on chromosome 12. The green bars in the junction track are the novel splice junctions, the blue bars are the current annotation of the splice junction. The red coverage track shows depth of sequence coverage, and the RefSeq track at bottom shows the current annotation for this portion of *MLL2*.

Greater than 100-fold more reads cross annotated splice junctions, relative to those reads crossing novel splice junctions. However, nearly 4% of the novel splice junctions in our dataset have more reads crossing them than reads crossing their annotated counterpart (Figure 2). Roughly 9% of the novel and the corresponding annotated splice junction have an equal number of reads crossing each splice junction. Over 87% of the annotated splice junctions have more reads crossing them relative to the corresponding novel splice junction. A novel exon in *MLL2* (ENSG00000167548) is a clear example where the novel junctions form a major transcript isoform of the gene (Figure 3).

### Characterization of the products of novel splice junctions

In order to better characterize the novel splice junctions, we categorized them into three types of events: novel exons, exon skipping, and alternate 3′ or 5′ splice sites. In this case, only novel events that occur within an annotated transcript (i.e. between two annotated exons) were considered. We identified 29,887 novel internal and terminal exons, 28,271 novel exon skipping events, and 21,757 novel alternate 3′ or 5′ splice sites that fit this criterion. Approximately half of these events occur in transcripts with an RPKM in the 5–25 range, consistent with the distribution of overall transcript abundance (Additional file 4).

**Figure 4:**
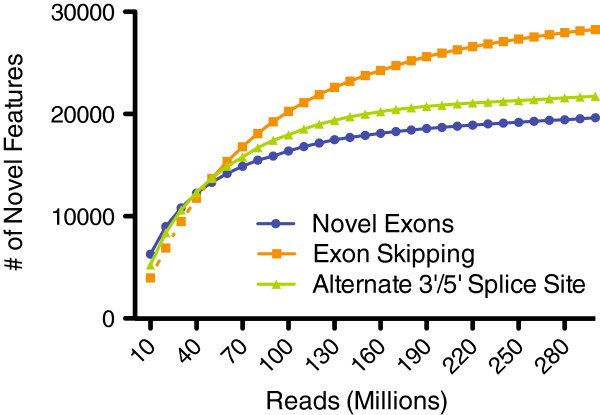
**Depth of coverage plots for the detection of novel splicing events in the human retinal transcriptome.** The percent of annotated features detected for 10 million read increments are shown. For novel exons and alternate 3′ or 5′ splice sites, the coverage plots nearly plateau at 300 million reads, suggesting that 300 million sequence reads provides good depth of sampling for these features. The slope of the exon skipping curve suggests that a majority of the exon skipping events have been detected, but that additional events could be detected with greater depth of sequencing.

Similar to the depth of coverage plot for the detection of annotated exons, we performed an analogous analysis for the detection of novel splicing events (Figure 4). A majority of the novel features are detected within the first 100 million reads (71-92%). However, analogous to the detection of annotated exons, as sequencing depth increases more novel features are discovered. Between 200 and 300 million reads, the percentage of new novel features detected is minimal, suggesting that we have sampled the transcriptome sufficiently.

**Figure 5:**
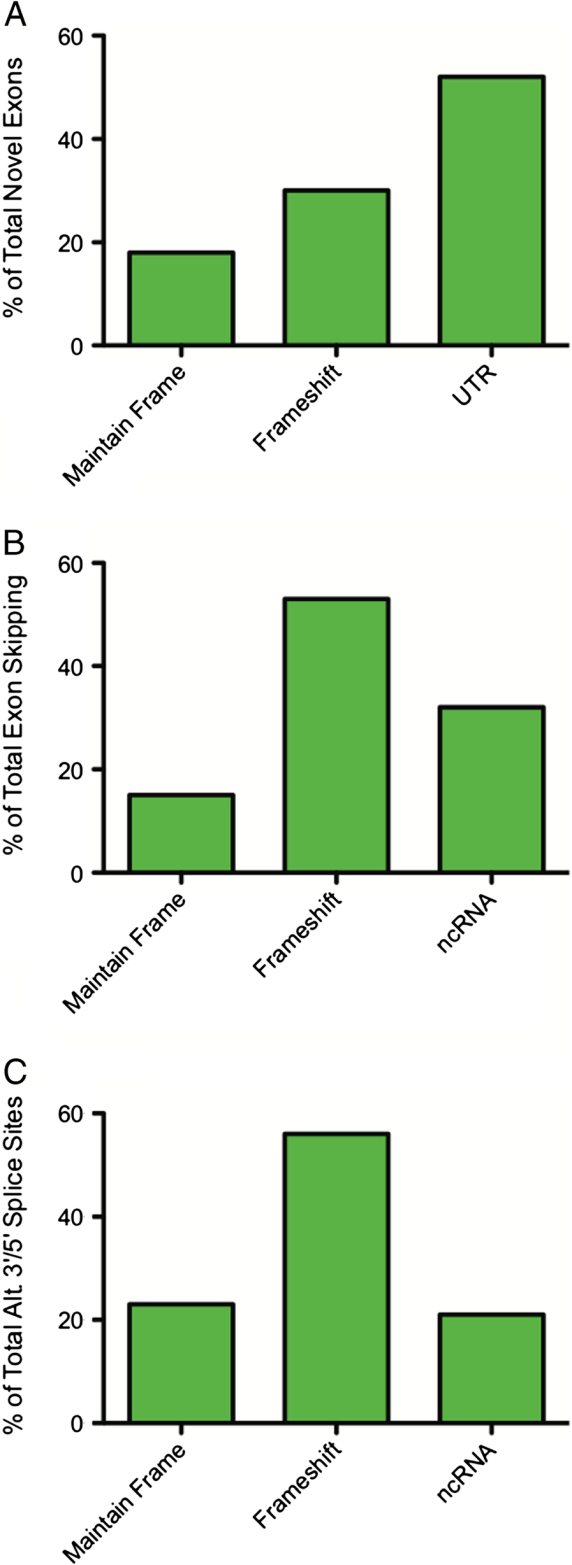
**The effects of novel transcript features/splicing on reading frame. A)** Of the 19,637 novel internal exons, 18% maintain an open reading frame (ORF), 30% result in a frameshift or contain a stop codon, and 52% are found within an untranslated region of the transcript. Of the 52% of novel exons that lie in an annotated UTR, 34% maintain an ORF (green bar) and 66% (gray bar) result in a frameshift or contain a stop codon. **B)** Of the 28,271 novel exon skipping events, 15% maintain an ORF, 53% result in a frameshift, and 32% are located in non-coding RNA (ncRNA). **C)** Of the 21,757 novel alternate 3′/5′ splice sites, 23% maintain an ORF, 56% result in a frameshift or contain a stop codon, and 21% are located in ncRNA.

We next set out to characterize the three types of novel events with respect to their effect on the coding regions of the annotated transcripts. The novel exons ranged in size from 8 to 482 bp with a mean of 118 bp. The 19,637 novel internal exons are found in 5,815 unique annotated transcripts. Eighteen percent are in-frame and maintain the coding sequence of the transcript (Figure 5A). Thirty percent of the novel exons cause frame-shifts, which are predicted to result in premature termination in the novel exons. The remainder (52%) form exons within the annotated untranslated regions (UTRs) of the transcripts. We have determined that 34% of the novel exons in UTRs maintain an ORF, while 66% result in a frameshift or contain a stop codon. In total, 36% of the 19,637 novel internal exons are in frame.

**Figure 6:**
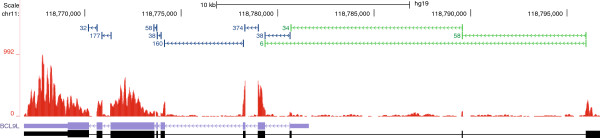
**An example of a novel terminal exon associated with an annotated transcript.** Through the use of the novel splice junctions (green bars) and coverage data (red), two novel exons were identified in this gene. The black bars below the gene annotation represent the novel gene model. In this example, a major isoform of this transcript includes the novel terminal exon.

Novel terminal exons are difficult to accurately identify because they can be ambiguous. A general analysis of the transcriptome identified over 34,000 novel terminal exons. To determine the false positive rate for this analysis, we visually inspected 500 randomly selected putative novel terminal exons using the following criteria: 1) if the terminal exon was part of a novel transcript, the transcript had to be 3 or more putative exons in length, 2) in all cases, the coverage of the terminal exon had to be consistent for at least 50 bp, and 3) a novel terminal exon within an annotated transcript must lie outside of the annotated UTR (An example novel terminal exon is shown in Figure 6). Applying these criteria to our dataset, we estimate that 30% (approximately 10,250 novel terminal exons) of the identified novel terminal exons are real.

Combining the novel internal and putative terminal exons, we have detected 29,887 previously unannotated exons. Relative to the combined 8 transcript reference tracks consisting of over 1 million exons, we have detected an additional 3% of exons. Of the annotated exons expressed in the retina, 4% of the exons detected in these analyses are novel. Based only on the novel internal exons (excluding novel terminal exons), these data contribute an additional 2.7 Mb of novel transcribed sequence to the annotated transcriptome. A list of the novel internal exons is provided as Additional file 5.

Exon skipping is reported to be the most common alternative splicing event [55]. We identified 28,271 novel skipping events found in 12,512 unique annotated transcripts in the retinal transcriptome. One or two exons were skipped the most frequently at 69% and 18% of the total novel exon skipping events, respectively (Figure 5B). Analyses of the ORFs for these events found that 15% maintained the ORF, 53% caused a frameshift, and 32% were located in annotated noncoding RNAs (ncRNA). On average 1.2 exons were skipped in the cases where the ORF was maintained, while an average of 2 exons were skipped when a frameshift was induced.

Alternate 3′ and 5′ splice sites can both add and remove sequence from a transcript [56,57]. We detected 21,757 novel alternate 3′ or 5′ splice sites in our dataset, which are found in 11,860 unique transcripts. Specifically, we detected 10,195 novel alternate 3′ splice sites and 11,562 novel alternate 5′ splice sites. We found that 23% of the novel alternate splicing events maintained an ORF (Figure 5C). Conversely, 56% caused a frameshift or introduced a premature stop codon. We also detected alternate 3′/5′ splice sites in ncRNA in 21% of the cases. The 8,720 events that resulted in new transcribed coding sequence are predicted to add 846 Kb of sequence to the transcriptome. There were 13,037 novel events that removed previously annotated transcribed sequence, resulting in a loss of 4.3 Mb of transcribed sequence in total. A list of the novel alternate 3′ and 5′ splice sites is provided in Additional file 4.

### Targeted enrichment and high-throughput validation

**Figure 7:**
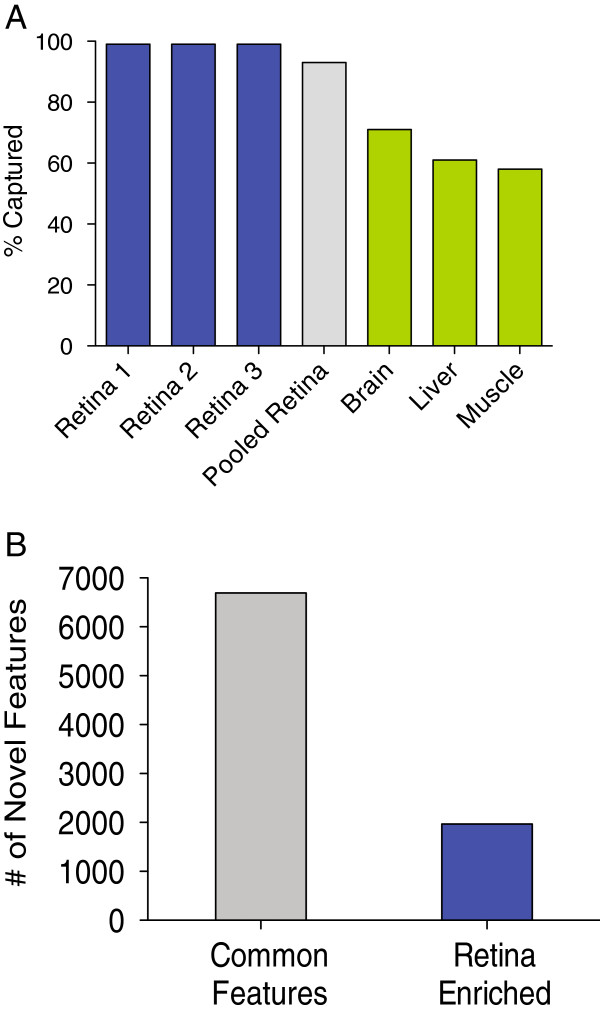
**High throughput validation of novel alternative splicing features.** From the novel features identified in the analyses of the human retinal transcriptome, 14,696 novel exons, exon skipping, and alternate 3′/5′ splice events were selected for capture using a custom Agilent SureSelect RNA Capture System. RNA samples from human retina, brain, muscle, and liver were enriched using baits designed against the novel features. The enriched samples were sequenced and aligned to the genome using the RUM pipeline. Following alignment, the samples were analyzed for the detection of the novel and annotated transcripts associated with the putative novel feature. **A)** 99% of the novel features detected by the RNA-Seq analyses were detected by the RNA capture in the individual retinal samples. 93% of all novel features were detected in a pooled library prepared from all three retinal RNA samples. Fewer of the novel transcript features were detected in RNA brain (71%), muscle (58%), and liver (61%). **B)** 6696 novel features were detected in all 6 RNA samples, while 1968 were detected only in the retina samples.

Recently, the technology used for targeted DNA capture and exome sequencing has been adapted for RNA capture [58]. We used this approach to investigate the reproducibility of the detection of novel transcript features identified in the RNA-Seq analyses, providing technical validation of our approach. For these studies, we selected a total of 14,696 putative novel splicing events from the retinal transcriptome data for capture, representing novel exons, exon skipping, and alternate 3′ and 5′ splice sites. We applied the capture technology to the original 3 retinal cDNA libraries individually, the three RNA samples combined and prepared specifically for the capture experiment, and to human skeletal muscle, brain, and liver RNA samples. Since not all 14,696 of the novel splicing events were found in all three samples, we performed individual capture analyses based on the specific set of novel events from each sample. Using sample-specific analyses, we were able to capture and validate 99% of the novel alternative splicing events in our target set (Figure 7A). In the pooled retinal sample, we validated 93% of the targets, while the brain, liver, and muscle samples validated at lower levels of 71%, 61%, and 58%, respectively. It should be noted that in these cases, unlike the original 3 retinal samples, the validation rate was determined by using the total capture set as reference, rather than the individual transcriptome as a reference. For every novel event in our capture set, we were also able to identify the corresponding annotated event. A list of the novel captured events and their corresponding rate of enrichment is provided in Additional file 6.

Alternative splicing among individuals is known to be variable [59-61]. As expected, the three retinal samples used for our analysis showed variability with regards to alternative splicing. This is likely a factor of gender, age, and technical factors such as post-mortem time to RNA isolation [62,63]. Among the novel events in the RNA capture set, nearly 7,000 were common within the three samples (Figure 7B). Of those 7,000 common novel events, nearly 2,000 were specific to these retinal samples, as they were not observed in the non-retinal samples. While these data are not a comprehensive analysis of the transcriptomes of all human tissues, the results suggest that these novel events are, at a minimum, enriched in the retina.

### Identification of novel genes

**Figure 8:**
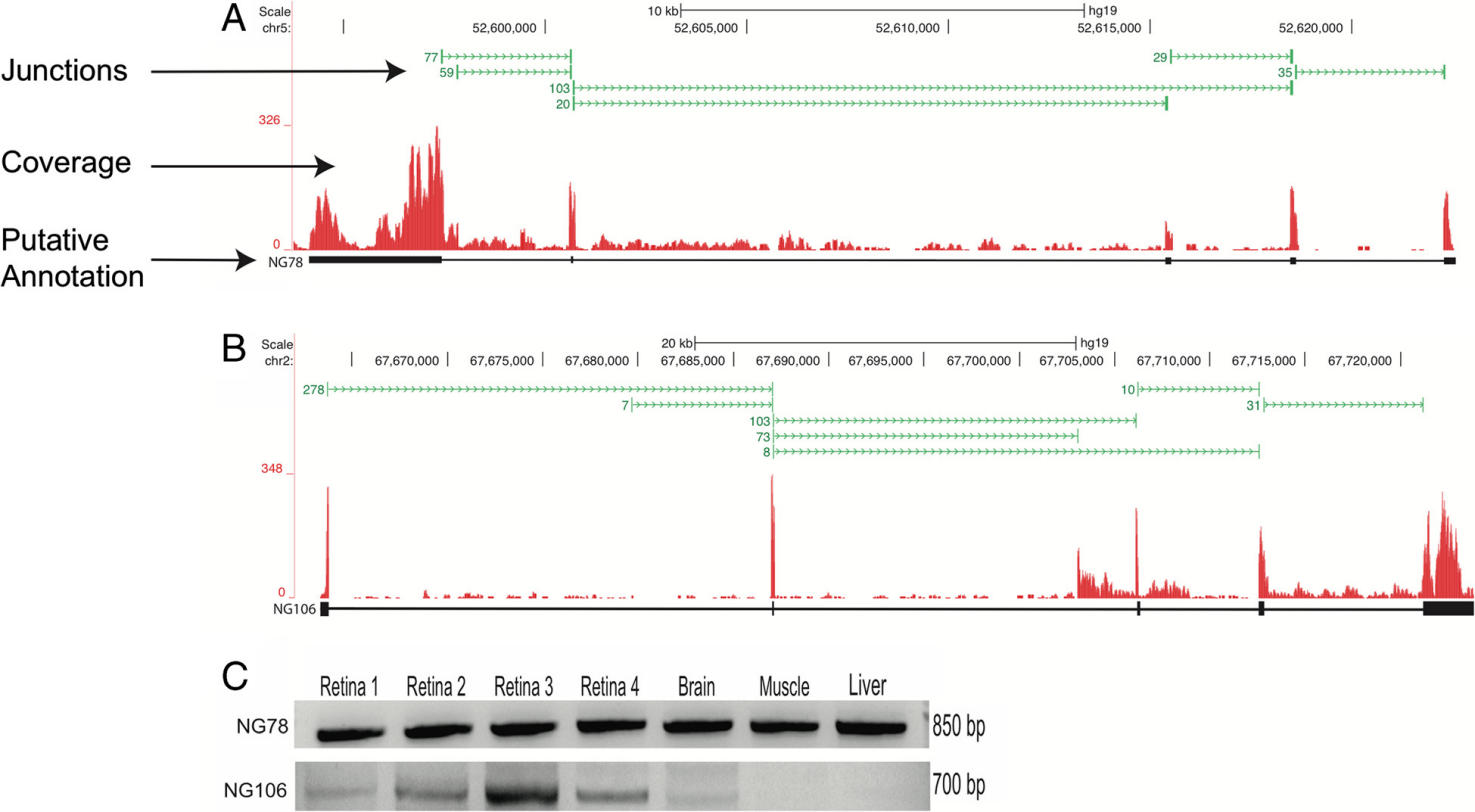
**Identification and tissue distribution of novel genes.** Using the novel splice junctions (green bars) and coverage data (red), transcripts with 3 or more novel exons located in intergenic regions were identified as putative novel genes (transcript models in black). **A)** An example of a five exon gene (NG78) with multiple isoforms that does not contain an ORF. **B)** An example of a six exon gene (NG106) that contains an ORF and is believed to be protein coding. **C)** Primers were designed in the putative terminal exon regions of the novel genes, and subject to RT-PCR and Sanger sequencing for validation of expression. NG78 was found in the original 3 retinal samples, an independent retinal sample (retina 4), as well as in the brain, liver, and muscle. NG106 was not found in the liver and muscle.

To further characterize the novel elements within the human retinal transcriptome, we also analyzed the data for transcripts derived from potential novel genes. For these analyses, we identified potentially novel genes based on an ENCODE-inspired definition that a “gene is a union of genomic sequences encoding a coherent set of potentially overlapping functional products” [64]. This definition is more broadly based and allows for inclusion of non-coding RNAs. Using this definition as a guideline, we developed the following rules to identify potentially novel genes as those that are: 1) intergenic, 2) contain 3 or more putative exons, and 3) are of sufficient read depth to provide consistent coverage across the exon (average read depth > 5). Using these criteria, we identified 116 potential novel genes (Additional file 7). We then manually curated the genes following the criteria described by Jia, et al. (2010) to determine their protein coding capacity [65]. Briefly, this method uses the length of the ORF (>80 amino acids), homology to known proteins, and conservation to other species as a foundation for determining the potential protein-coding capacity of a gene. We found that 16 of the genes contained an ORF that was greater than 80 amino acids in length. For the 97 genes with an ORF less than 80 amino acids, we did not identify homology with proteins known in human and non-human primates. For these 97 novel genes that had ORFs of less than 80 amino acids in length and were not found to have homology with known proteins, we classified them as encoding potential lincRNAs [24]. Figure 8A depicts a novel gene that we believe to encode a lincRNA. It is composed of 5 exons with an estimated length of 2184 bp, and no ORF. Figure 8B is an example of a gene that is composed of 6 exons with an estimated length of 925 bp, and an ORF of 90 amino acids.

### Validation of novel genes

We were able to validate up to 99% of the novel splicing events detected in the RNA-Seq data in the RNA capture experiment, suggesting that the identified novel genes are real. In addition, we selected 10 of the potential novel genes for further validation using RT-PCR and Sanger sequencing. To do this, we designed primers to the putative terminal exons of the novel genes, without preference to the abundance of the isoforms that contained these exons. These novel genes varied from 925 to 10,205 bp in length, represented by 3 to 7 exons (Additional file 8). RT-PCR from human retinal RNA, followed by sequencing, validated the expression and full-length sequence of all 10 of the novel genes.

To evaluate the distribution of expression of these novel genes, we also tested the human brain, liver, and muscle RNA samples for expression of the 10 novel genes by RT-PCR. We found that 9/10 of the putative novel genes were expressed in all tissues tested, with the only exception being NG106, for which as noted in Figure 8B expression was not detected in the muscle and liver samples.

## Discussion

### A more complex transcriptome

The data presented here indicate that the retinal transcriptome is more complex than previously understood in at least two ways. First, the RNA-Seq analyses of human retinal RNA samples detected 75% of previously annotated exons, based on the combined annotation track used for these studies, which includes over 1 million unique exons. This is a notable increase over earlier estimates of the number of genes expressed in the retina, which suggested that 26,355 transcripts were expressed in this tissue [32]. This diversity of gene expression is not entirely surprising, however, given the cellular diversity of the retina, with at least 60 functionally distinct cell types [1]. Our results are comparable to those found in the RNA-Seq analyses of the ENCODE project [54]. In those studies, consortium investigators used RNA-Seq to analyze the transcriptomes of 15 cell lines, and determined that 85% of the annotated exons in the Gencode V7 transcript database were expressed in the aggregate data. As shown in Figure 1B, we identified 80% of the annotated exons found in the same reference transcriptome.

Our data suggest that the current annotations may under represent the full extent of expressed exons in the human genome. This is likely due to the conservative approach used to build an annotation databases such as the CCDS [42]. This conservative approach provides a very high quality annotation set at the expense of non-coding regions of protein coding genes, as well as genes that encode non-coding RNAs. The lack of transcriptomes that have been fully characterized in a wide variety of tissues is likely to be another factor for incomplete annotation databases. For example, we have identified over 29,000 novel exons in a single tissue suggesting that RNA-Seq studies in other tissues is warranted to truly understand the complexity of gene expression at an organismal level [20,23,56,58,66-70].

We report here the first RNA-Seq based characterization of the human neural retina transcriptome, although there have been previous RNA-Seq analyses of the mouse retinal transcriptomes [35,71,72]. Brooks, et al. (2011) noted that between 16,000 and 34,000 transcripts are expressed in the mouse retina, which is in line with earlier EST and microarray based estimates in the human retina [32,71]. From a functional and disease standpoint, Gamsiz, et al. (2012) determined that 24% of the retinal genes are alternatively spliced and genes implicated in IRDs are among some of the most highly expressed [72]. While these data are interesting, they lack sufficient read depth to fully cover the retinal transcriptome. We previously reported that not only were 100 million reads necessary to fully cover the mouse retinal transcriptome, but tens of thousands of novel splicing features can be identified as well [35].These data combined provide detailed insight into the mouse retinal transcriptome, but it should also be noted that there is considerable variation in gene expression and alternative splicing among species, thus increasing the importance of a thoroughly characterized human transcriptome [57,73,74].

At present, it is not possible to determine how the complexity of the retinal transcriptome compares to other tissue types, since this is the first reported use of a complete annotation track for transcriptome analyses. Further, other reported transcriptome analyses may overestimate the number of novel features discovered due to the incompleteness of the reference annotation tracks used [20,23,51,53,75]. For example, we have incorporated the lincRNA annotation track into our study and detected approximately 35% of the more than 50,000 known lincRNA exons, thus preventing incorrect identification of 17,500 lincRNA exons as novel [38]. Kim, et al. (2012) identified as many as 1007 novel transcripts using Refseq, Ensembl, UCSC and Vega as their annotation database, however a quick review of some of the novel genes shows that they are lincRNAs, while others partially overlap annotated protein coding genes [23].

Questions have been raised about the biological importance of novel transcript features detected at low abundance by RNA-seq. For example, it has been suggested that “noisy,” or inaccurate splicing can account for rare novel splicing events detected by RNA-Seq [52,76]. RNA-Seq studies evaluating novel splice site location and conservation of the splice junction have shown that splicing errors occur at a rate of 0.7% [52]. The authors of this study conclude that low abundance novel splice junctions may be due to erroneous splicing because they are often enriched around annotated splice junctions, suggesting that the spliceosome misses its intended target. In addition, because many of these events are not evolutionarily conserved, it has been suggested that splicing errors render the resulting transcript isoforms non-functional due to nonsense mediated decay (NMD) [77,78].

A large majority (97%) of the retinal transcriptome is found to be expressed at a low to mid-level range (25 or fewer transcripts per cell). Djebali, et al., 2012, noted 80% of the detected transcripts existed as 1 or fewer copies per cell in the 15 cell lines studied in the ENCODE project, whereas 24% of the transcripts in the neural retina transcriptome exist at this level [54]. This can likely be explained by the cellular diversity of the neural retina [1]. Approximately 75% of the novel features are found in transcripts expressed between 5–100 transcripts per cell. This is interesting given this class represents 47% of the expressed transcripts, the other 48% are expressed at fewer than 5 transcripts per cell, yet contain only 16% of the novel features.

While up to 15% of the novel features in the retinal transcriptome compose the major isoform, or are expressed at the same level as the annotated isoform, the remaining 85% constitute the minor isoform. The analyses reported here suggest that at least a portion of low abundance novel transcript features detected in the retina produce novel protein-coding transcripts, which may have functional importance in the retina. Since the NMD machinery is thought to help regulate expression of transcripts with premature stop codons, we hypothesize that novel transcript isoforms which maintain an ORF are likely to encode proteins and be functional [77,79]. We found that 15-36% of the novel splicing events detected in our dataset maintain the ORFs of the transcripts they affect. It has also been suggested that alternative splicing events which do not maintain an ORF may play roles in the regulation of gene expression and genomic evolution [78,80,81]. Additional studies of the novel transcript isoforms identified will be needed to test these hypotheses.

The more comprehensive human retinal transcriptome described here will likely be beneficial to studies of the genetics of retinal dystrophies and other inherited retinal disorders. Novel exons in the *BBS8* and *RPGR* genes were discovered to harbor mutations that lead to IRDs [12,13]. Our analyses identified 206 novel exons in 99 of the 191 known IRD disease genes. The novel features in the retinal transcriptome can also be applied to genes that cause disease in other tissues. For example, we have identified novel exons in *MLL2*, which harbors mutations known to cause Kabuki Syndrome [82,83]. Our transcriptome data offers a unique resource in which genes can be quickly screened for novel features that can be incorporated into genetic analyses. To fully utilize this resource, we have included as separate files lists of all novel internal exons identified in our analyses (Additional file 2). These data, in combination with the coverage and splicing data viewable on UCSC, provide easy access for researchers to identify novel splicing features in genes or loci of interest.

RNA-Seq has become the standard approach for studying the transcriptomes in a variety of organisms and the changes in gene expression that occur in a wide range of diseases [84-88]. Many RNA-Seq studies have reported the identification of thousands of novel features with the assumption that a certain percentage of these are real, based on low-throughput validation studies of selected transcript variants via RT-PCR and sequencing [23,66,67,75,86,89]. In a previous study, we had identified over 12,000 novel features in the mouse retina, and attempted to validate 75 (0.63%) of the features [35]. While we were able to validate 64% of the 75 novel features, this question remains: can validation of a small number of novel transcript features be extrapolated to the thousands of novel transcript variants?

One approach to answer this question is to validate the results of RNA-Seq studies on a large scale. High-throughput targeted RNA capture is a novel approach that offers enhanced specificity and scale over more traditional validation methods such as RT-PCR. Where RT-PCR uses primers designed to a specific region of a transcript, targeted RNA capture uses hybridization baits that are tiled over a specific region of interest. In our study, we used 3X tiling of baits for each of the 14,696 novel features to provide specificity for the targeted transcript features. The data obtained show that 99% of the novel transcript features identified in the RNA-Seq analyses were detected in the RNA capture experiments. This result provides robust technical validation of the RNA-Seq analyses. To our knowledge, this is the first report of such a validation of RNA-Seq data. Because the RNA capture set incorporated 19% of the novel transcript features detected by RNA-Seq, we believe these data can be more reliably extrapolated to the whole RNA-Seq dataset.

The RNA capture experiments demonstrated that 7,000 of the novel transcript features studied were shared between all three retinal RNA samples, further confirming their presence in the human retinal transcriptome. Approximately 2,000 of these novel transcript features were not detected in the RNA capture experiments using human brain, liver and muscle RNA, suggesting that these splicing alterations may be specific to the retina. Extrapolation of these data indicate that up to 2.5% of the novel transcript features detected in the RNA-seq analyses may be retina specific, with the potential to provide insight into retinal biology.

As a corollary, the RNA capture experiments also showed that 63-71% of the novel transcript features identified in the retina were shared with brain, liver and muscle. These results provide biologic validation for these novel transcript features, with their detection in other tissues. They also suggest that transcriptome annotation in other tissues such as brain, liver and muscle may also be incomplete, since the non-redundant reference transcriptome we used is not tissue-specific. In total, these data suggest that RNA-Seq experiments analyzed by the RUM pipeline produce high quality and reliable results that are applicable to other tissues [35]. This further supports the conclusion that RNA-Seq experiments do not require large-scale validation once the experiment and analysis pipeline have been thoroughly validated. We suggest that it would be both worthwhile and informative to apply a similar comprehensive empiric validation methods to other analysis pipelines.

Finally, we identified 116 potential novel genes. These novel genes are located in regions of the genome that are currently annotated as intergenic, and varied in the number of exons, length, alternative isoforms, and coding potential. In an attempt to be conservative with our definition of a novel gene, we identified only those that were three or more putative exons, of which all exons of the putative gene were completely intergenic. A majority (81%) of the novel genes identified showed little to no coding potential. We have identified these genes as encoding putative lincRNAs. Many potential lincRNAs have also been identified in transcriptome analyses of other tissues [84]. lincRNAs are typically classified as non-coding RNAs that are longer than 200 bp, intergenic, spliced, and polyadenylated [24]. Functionally, lincRNAs are best known for their role as regulators of gene expression through associations with chromatin modifying complexes [90-92]. Additional studies will be required to investigate the function of the putative novel genes lincRNAs identified by these studies in the retina.

## Conclusions

Novel transcriptome features, both alternative splicing of annotated genes and transcription of novel genes, is more abundant than previously understood in the human neural retina. Further, by identifying tens of thousands novel alternative features, and validating a significant portion of them, these transcriptome data have increased our understanding of transcription, in general. Ultimately, these data have the potential to influence disease research. By elucidating the specific isoforms present in the retina, disease genes can be more appropriately studied.

## Methods

### RNA-seq library preparation and sequencing

Total RNA samples from normal human retina from a male and two females of ages 42, 44, and 46 years old, respectively were purchased from Biochain. No post-mortem time information was available, although all samples had a RIN between 7–8, suggesting the RNA was slightly degraded. The individuals from which these samples were obtained were not reported to have ocular disease, although detailed medical information is not available. RNA-Seq libraries were prepared from 5 μg total RNA following a modified Illumina mRNA-Seq protocol (Illumina). Briefly, mRNA was purified using oligo-dT beads (Invitrogen). The purified mRNA was then fragmented for 2 minutes at 94°C on a thermalcycler with the addition of 2 μl of 10X fragmentation buffer (1 mM ZnCl_2_, 1 mM Tris–HCl, pH 7.0). The reaction was stopped with the addition of 4 μl of 100 mM EDTA, pH 8.0. First strand cDNA was synthesized using SuperScript II reagents following manufacturer’s instructions (Invitrogen). The remaining steps: second strand cDNA synthesis, end-repair, monoadenylation, and adapter ligation were performed following Illumina’s mRNA-Seq protocol. All reagents, excluding paired-end adapters, were purchased from New England Biolabs. Paired-end adapters and PCR primers were purchased from Illumina. cDNA libraries were size selected using a 2% agarose gel and fragments between 300–350 bp were selected. Size selected samples were then PCR amplified using 15 cycles. cDNA library quantity and quality were determined using DNA 1000 chips on a Bioanalyzer 2100 (Agilent). Each of the three samples were clustered at 10 pM in individual lanes of a flow cell. 101 bp paired-end sequencing was performed on an Illumina HiSeq 2000. For quality control, PhiX was spiked into each sample at 1% of the sample concentration.

Additional normal human brain, muscle, liver, and the retina samples described above were prepared for targeted capture using a modified Nextera DNA library preparation protocol (Illumina). Total RNA for all samples was purchased from Biochain. To prepare cDNA libraries, 185 ng of total RNA was first converted to double stranded cDNA. First strand cDNA was prepared using Superscript III with anchored oligo-dT primers according to manufacturers protocol (Invitrogen). Second strand cDNA was produced using RNase H, DNA polymerase I, dNTPs, and Second Strand buffer (New England Biolabs). The reaction was incubated for 2.5 hours at 16°C. Following the incubation, the samples were purified using Clean and Concentrator-5 spin columns (Zymo). The samples were eluted with 25 μl of molecular biology grade water. Fragmentation, PCR amplification, and bead purification of the cDNA libraries was performed according to the Nextera protocol. Finally, the libraries were size selected using a 1% agarose gel and fragments between 400–600 bp were selected.

### Alignment and post-processing of RNA-seq reads

All data was aligned using the RNA-Seq Unified Mapper (RUM v1.10) pipeline using the default settings against the hg19 genome and no transcriptome database. Due to aligning the data without a transcriptome, the files containing the splice junctions (both novel and annotated) were generated manually using the make_RUM_junctions_file.pl script that is downloaded with the RUM pipeline. The annotation file used to generate the splice junctions was a non-redundant transcriptome database generated from the 8 empirically-determined transcriptome annotation tracks (UCSC, Refseq, Vega, Aceview, Ensembl, Gencode V7, CCDS and lincRNA) [32,42-48].

### Identification of novel features

Novel exons, alternate 3′/5′ splice sites, and exon skipping events were identified using the splice junction file generated using RUM. The splice junction files contain both novel and annotated splice junctions based on the 8-track annotation file, which can be parsed to identify each of the novel features. Novel exons were determined by pairing a left coordinate of a novel splice to the right coordinate of a novel splice junction, if they are within 15–650 bp of each other. Novel alternate 3′/5′ splice sites were identified where either the 3′ or 5′ coordinate is shared with an annotated exon and the novel site is 15–350 bp upstream or downstream of the corresponding site of the annotated exon. Novel exon skipping events were determined by identifying novel splice junctions that share are within annotated transcripts and span at least one annotated exon.

### Determination of open reading frame

Custom scripts were developed to determine the open reading frames of novel features within annotated transcripts. For each novel feature, the script determined the location of the feature within the annotated transcript found in the 8-track transcriptome. Each type of novel feature (novel exons, exon skipping, and alternate 3′/5′ splice sites) first required the addition or removal of sequence from the annotated transcript sequence, depending on the novel feature. Using the CDS start coordinate, the novel transcript isoforms were translated and the position of the stop codon determined.

### Targeted RNA capture and high-throughput validation

Using the RUM_Unique and splice junction files, 14,696 novel internal exons (novel exons found between two annotated exons), exon skipping, and alternate 3′/5′ splice sites were selected for high-throughput validation. Selection of novel features was dependent on a few criteria: 1) novel exon and alternate 3′/5′ slice site length was between 15–400 bp, 2) the novel features had to be flanked by at least 50 bases of annotated sequence, and 3) the total region to be captured (novel + annotated) had to be between 300–500 bases in length. Baits to the regions of interest were generated using Agilent’s eArray software. Blockers designed specifically to the Nextera adapters were provided separately by Agilent. Capture was performed following the manufacturer’s protocol on the human retina, brain, muscle, and liver samples. Sequencing and alignment was performed as described above. However, the data was aligned using both the hg19 genome and the 8-track transcriptome.

### Identification and coding potential of novel genes

The list of novel exons was parsed to identify exons that were intergenic based on the 8-track transcriptome. This list was used to manually determine if they belonged to a putative novel gene. The criteria used to determine if an exon belonged to a novel gene were: 1) based on splice junction data, the novel exon was not connected to another exon that overlapped an annotated gene, 2) at least 3 novel exons were connected to form a novel gene, and 3) there was sufficient coverage data (average read depth of 5).

Open reading frames of the novel genes were determined for all six frames. Homology for ORFs that contained both a methionine as a start codon and an in-frame stop codon were determined using BLAST [93].

### Validation of novel genes

First-strand cDNA was produced from the 3 human retinal tissues, brain, liver, and muscle used in the previous transcriptome studies, as well as a fourth retinal tissue (Biochain) using SuperScript III following the manufacturer’s instructions (Invitrogen). Primers were designed to the putative terminal exons of the novel genes and amplified using Phusion polymerase (New England Biolabs). The PCR products were visualized on a 1% agarose gel. Amplicons of the expected size were excised and purified using the Zymoclean Gel DNA Recovery Kit (Zymo). The products were Sanger sequenced, and confirmation of the novel gene was performed by aligning the output against the retinal transcriptome data hosted at UCSC using BLAT.

## Abbreviations

IRD: Inherited retinal degeneration; lincRNA: Long intergenic non-coding RNA; UCSC: University of California, Santa Cruz; BLAT: Blast-like alignment tool; ORF: Open reading frame; RUM: RNA-Seq Unified Mapper; NMD: Non-sense mediated decay.

## Competing interests

The authors declare no conflict of interest.

## Authors’ contributions

MHF and EAP conceived and designed the experiments. MHF, JAW, and GRG analyzed the data. MHF, MBC, and MES performed the experiments. MHF and EAP wrote the manuscript. All authors read and approved the final manuscript

## Supplementary Material

Additional file 1 **Non-redundant reference transcriptome.** Complete annotation database consisting of all 8 human databases. Click here for file

Additional file 2 **Distribution of expression of annotated transcripts.** Graph of the RPKM values of all expressed transcripts. Click here for file

Additional file 3 **Description of data on UCSC Genome Browser.** Description of transcriptome data on the UCSC Genome Browser. Click here for file

Additional file 4 **Distribution of transcript abundance containing a novel feature.** Graph of the RPKM values of annotated transcripts which contain a novel feature. Click here for file

Additional file 5 **List of novel genes identified in the human retinal transcriptome.** Novel internal exons and alternate 3′/5′ splice sites. List of novel internal exons and alternate 3′/5′ splice sites. Click here for file

Additional file 6 **Table of validated novel genes.** Validation of novel features. List of novel alternate splicing features used for capture/validation and corresponding enrichment. Click here for file

Additional file 7 **Novel genes.** List of novel genes identified in the human retinal transcriptome. Click here for file

Additional file 8 **Validated novel genes.** Table of validated novel genes. Click here for file

## Data access

We have made our data publically available in two formats. First, we have uploaded our RNA-Seq data to GEO (GSE40524). We have also made our data viewable on the UCSC Genome Browser (http://oculargenomics.meei.harvard.edu/index.php/ret-trans/110-human-retinal-transcriptome). A description of the browser features can be found in Additional file 3.
