# Supplementary figures and images for: Transcriptome analyses of the human retina identify unprecedented transcript diversity and 3.5 Mb of novel transcribed sequence via significant alternative splicing and novel genes
Source: BMC Genomics. 2013 Jul 18;14:486. doi: 10.1186/1471-2164-14-486 (PMC3924432; doi:10.1186/1471-2164-14-486)

% of Total Detected Transcripts

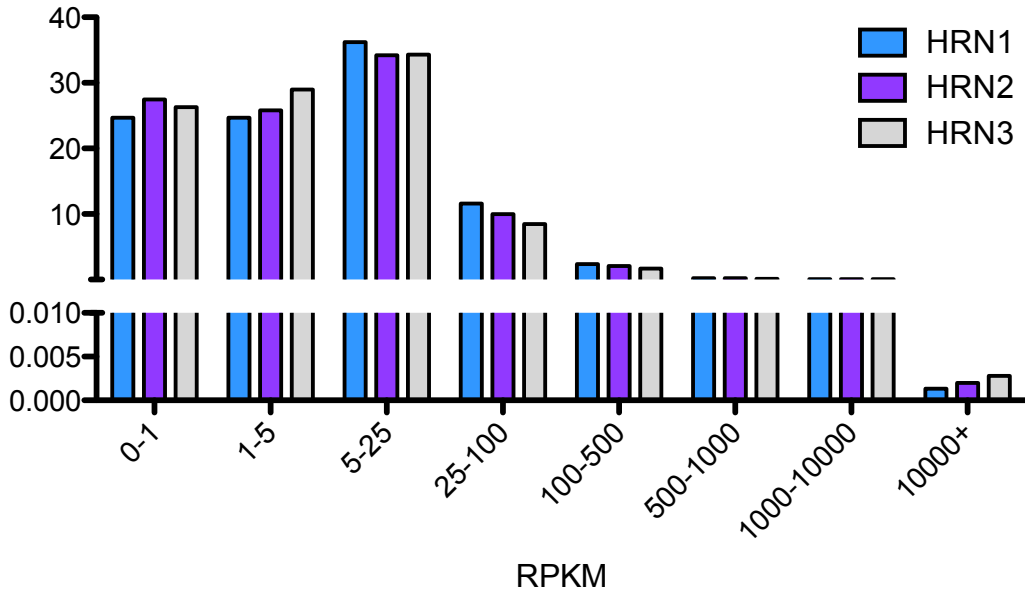

Supplement: Additional file 2 — Distribution of expression of annotated transcripts. Graph of the RPKM values of all expressed transcripts. [file 1471-2164-14-486-S2.pdf]

% of Total Detected Transcripts

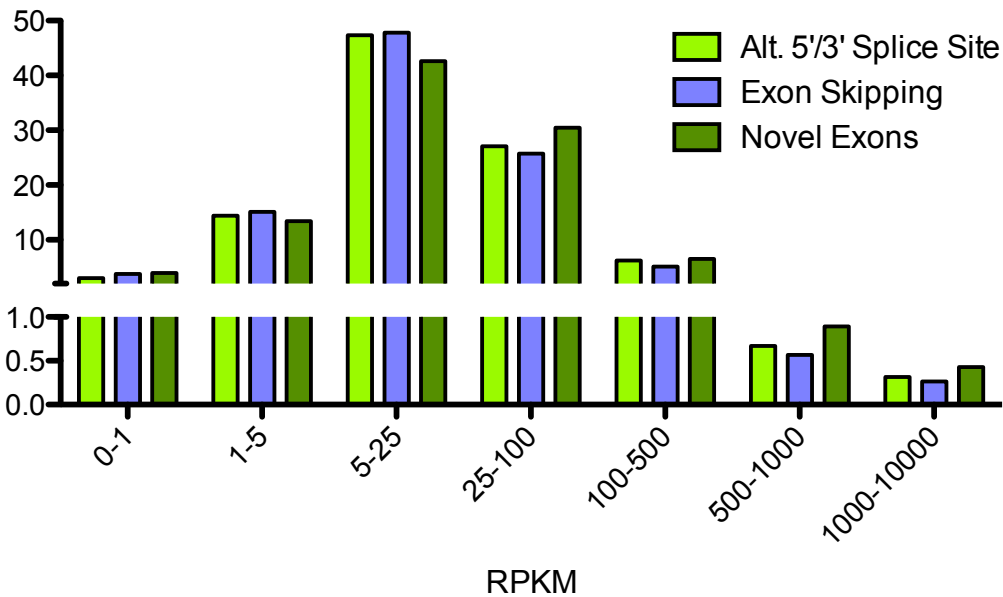

Supplement: Additional file 4 — Distribution of transcript abundance containing a novel feature. Graph of the RPKM values of annotated transcripts which contain a novel feature. [file 1471-2164-14-486-S4.pdf]
