# Supplementary material for: Transcriptome analyses of the human retina identify unprecedented transcript diversity and 3.5 Mb of novel transcribed sequence via significant alternative splicing and novel genes
Source: BMC Genomics. 2013 Jul 18;14:486. doi: 10.1186/1471-2164-14-486 (PMC3924432; doi:10.1186/1471-2164-14-486)
Supplement: Additional file 3 — Description of data on UCSC Genome Browser. Description of transcriptome data on the UCSC Genome Browser. [file 1471-2164-14-486-S3.docx]

**Additional File 3.** Description of the RUM output of the human retinal transcriptome in the UCSC Genome Browser available at <http://oculargenomics.meei.harvard.edu/index.php/ret-trans/110-human-retinal-transcriptome>. **A)** Current gene annotation. The colored boxes represent the exons, both coding and untranslated regions. The connecting lines and arrows denote introns and coding directionality. **B)** The coverage track is a graphical representation of the alignment of the sequencing reads to the genome and transcriptome. **C)** Read depth. This number is not an average across the feature, but rather it represents the highest read depth in the feature window. **D,E)** The junction track is a graphical representation of sequencing reads that cross splice junctions, with blue depicting annotated junctions and green depicting novel junctions. The vertical blue lines denote the point at which the read is split and mapped to two distinct genomic locations. The size of the vertical blue line is arbitrarily set to 50 bp and does not represent the number of bases that align to each side of the junction. The connecting line denotes the region between the alignment of the read (intron). The samples were prepared using a non-strand-specific protocol, so the arrows on the connecting lines of the reads crossing splice junctions are arbitrarily set to the plus strand, and are not informative. **F)** This number represents the number of reads that cross a distinct splice junction.
