# Supplementary material for: Transcriptome analyses of the human retina identify unprecedented transcript diversity and 3.5 Mb of novel transcribed sequence via significant alternative splicing and novel genes
Source: BMC Genomics. 2013 Jul 18;14:486. doi: 10.1186/1471-2164-14-486 (PMC3924432; doi:10.1186/1471-2164-14-486)
Supplement: Additional file 8 — Validated novel genes. Table of validated novel genes. [file 1471-2164-14-486-S8.docx]

**Additional File 8**. Table of validated novel genes including the chromosomal coordinates, putative exons, approximate transcript length, and primer sequences used for validation.

Table S1. Validated novel genes

| Name | Coordinates | # of Exons | Length (Approx.) | Forward Primer | Reverse Primer |
| --- | --- | --- | --- | --- | --- |
| NG 3 | chr11:61,809,393-61,860,591 | 7 | 10,205 | TCCTGCATGGTTTTCTTGAGTTGTC | AAATATACTCTTTTGTCTTTATCTTTTACTCCTGC |
| NG 4 | chr16:50,423,670-50,431,371 | 3 | 1508 | CACCTGGTCCTCAGCCCTTTTCTC | ATAACGCCCAAATCAACAAATCTGC |
| NG 18 | chr11:115,497,665-115,518,521 | 3 | 2305 | GTAAGCTGGCTAAGGCTCTTAGAAGGC | CAGATGTAAAATAGAAATCTAATGATCATACCTCTCTC |
| NG 51 | chr2:126,451,088-126,575,182 | 5 | 2684 | TGGGTGTTTACCTTTAGAGCTTCAGTTTC | TCTAGCAGACCAGCAGTAGACACCAGAC |
| NG 52 | chr2:137,108,175-137,456,001 | 6 | 2028 | CCATTATAAAAGGGCATGGTAGAGAAAGC | AGATGAGAAAGAACCAGCACAACAACTC |
| NG 66 | chr4:31,578,058-31,606,670 | 3 | 544 | CAGCATGGCTAGAACAAAACAGGC | TGTCTTGACAAAAGAACAAAAGAAAGGATTC |
| NG 78 | chr5:52,950,000-52,625,000 | 5 | 1050 | CACGCTCCCTGTGTATTTCTGTCTTCTC | AAATGCTTTAGTTGGTGGGTGCTCC |
| NG 34 | chr6:80,466,595-80,505,274 | 5 | 5520 | TGTTCTTAATATCAGTGTGCTCACTCGTCC | CCAGCGGCCGCCAAAGAC |
| NG 46 | chr2:67,634,892-67,750,927 | 7 | 5682 | GAAAACAAGGCACTGGGAAGAACATC | GCTCTTTCCAAGCCATAGTCTACACAGTC |
| NG 106 | chr13:31,981,974-32,050,198 | 7 | 925 | CATTGAAGCCACATGTGCAGACCAC | AGGACTCCTCGTACCTGAAATGATGG |
